# Ultra-Stretchable and Skin-Conformal Piezoelectric Electronic Skin for Self-Powered Human Motion Monitoring

**DOI:** 10.3390/s26154781

**Published:** 2026-07-28

**Authors:** Jingchao Yuan, Junbin Yu, Jian He, Jiliang Mu, Xiaojuan Hou, Xiujian Chou

**Affiliations:** Science and Technology on Electronic Test and Measurement Laboratory, North University of China, Taiyuan 030051, China; b20220628@st.nuc.edu.cn (J.Y.); drhejian@nuc.edu.cn (J.H.); mujiliang@nuc.edu.cn (J.M.); houxiaojuan@nuc.edu.cn (X.H.)

**Keywords:** piezoelectric electronic skin, ultra-stretchable sensor, skin-conformal, self-powered monitoring, human motion recognition

## Abstract

Soft piezoelectric electronic skins are expected to work reliably under repeated joint bending, where both stretchability and stress transfer efficiency are important. In this work, a BaTiO_3_/PDMS piezoelectric electronic skin, denoted as BPPE-skin, was prepared by a blade-coating method. Rather than only increasing the amount of piezoelectric filler, this study focuses on how the PDMS matrix composition affects the mechanical deformation and electrical response of the composite. The PDMS base-to-curing-agent ratio was adjusted from 5:1 to 30:1, and the BaTiO_3_ loading was varied from 10 to 90 wt%. Tensile tests show that increasing the base-to-curing-agent ratio reduces the elastic modulus and improves stretchability, while excessive softening weakens effective stress transfer. As a result, the voltage output reaches its highest value at a 10:1 ratio. Increasing the BaTiO_3_ content further enhances the output, mainly because more active piezoelectric particles participate in electromechanical conversion. The optimized film, containing 90 wt% BaTiO_3_ with a 10:1 PDMS ratio, maintains good flexibility and can be attached conformally to different body joints. Tests on the knee, elbow, wrist, and fingers produce distinct voltage patterns during bending motions, indicating the potential of BPPE-skin for wearable, self-powered human motion monitoring.

## 1. Introduction

Wearable electronic skins have become an important class of soft sensing platforms for human motion monitoring, rehabilitation assessment, personalized healthcare, sports analysis, and human–machine interaction. Unlike rigid sensors, skin-mounted devices must operate on curved, soft, and continuously moving body surfaces, where bending, stretching, twisting, and local compression often occur simultaneously. For this reason, an effective wearable sensor should combine mechanical compliance with stable signal output. If the sensing layer cannot conform closely to the skin, interfacial gaps and relative sliding may generate motion artifacts and reduce the reliability of the measured signals [1,2,3,4,5,6,7,8,9]. Among various sensing mechanisms, piezoelectric electronic skins are attractive because they can directly convert dynamic mechanical deformation into electrical signals, allowing self-generated signal output without a continuous external power supply [10,11,12,13,14,15]. This feature is particularly useful for lightweight wearable systems and distributed motion monitoring. However, most high-performance piezoelectric ceramics are intrinsically rigid and brittle, which makes them difficult to integrate with human joints that experience large and irregular deformation. To overcome this limitation, piezoelectric ceramic fillers, such as BaTiO_3_, have been embedded into elastomeric matrices to form flexible piezoelectric composites. In such systems, BaTiO_3_ provides the active piezoelectric phase, while the polymer matrix contributes stretchability, conformability, and processability [16,17,18]. Compared with purely polymeric piezoelectric films, ceramic–elastomer composites also offer a practical route to combine relatively high piezoelectric activity with simple fabrication and large-area formability.

Polydimethylsiloxane (PDMS) is one of the most widely used elastomers in wearable sensors because of its low modulus, high deformability, biocompatibility, chemical stability, and simple processing [19,20,21,22]. In recent years, PDMS-based piezoelectric composites have been extensively studied to improve the output performance and mechanical adaptability of flexible sensors and nanogenerators. Reported strategies include increasing the content of piezoelectric fillers, improving the dispersion of ceramic particles, constructing filler networks, aligning particles under external fields, designing multilayer structures, and introducing surface microstructures [23,24,25,26,27,28]. These approaches have effectively improved charge generation, stress concentration, or contact deformation in flexible piezoelectric devices. Several BaTiO_3_-polymer-based wearable sensors have also been reported for human motion monitoring. For example, Li et al. developed a flexible lead-free Ba_0.94_Sr_0.06_Sn_0.09_Ti_0.91_O_3_/PDMS composite for self-powered human motion monitoring, where the improvement mainly originated from the compositional modification of the BaTiO_3_-derived piezoelectric ceramic phase [29]. Yu et al. reported a skin-conformal BaTiO_3_/Ecoflex-based piezoelectric nanogenerator, in which the highly stretchable Ecoflex matrix contributed to conformal attachment to human skin and motion sensing [30]. Fernandez et al. investigated a capacitive BaTiO_3_-PDMS hand-gesture sensor and further analyzed its sensing mechanism and signal classification using machine learning [31]. These studies demonstrate the feasibility of BaTiO_3_-polymer composites for wearable sensing applications. However, previous studies mainly focused on ceramic-phase modification, active filler enhancement, soft-matrix conformability, capacitive sensing mechanisms, device-structure optimization, or signal classification strategies. Nevertheless, the elastomeric matrix itself is often treated as a passive supporting phase, and its influence on electromechanical transduction has not been sufficiently clarified. In a BaTiO_3_/PDMS composite, the matrix determines how external strain is distributed and how efficiently stress is transferred to the embedded piezoelectric particles. A matrix with excessive stiffness may restrict device deformation and weaken skin conformability, whereas an overly soft matrix may dissipate part of the applied mechanical energy through viscoelastic relaxation, thereby reducing effective stress transfer. Therefore, an appropriate matrix compliance is required to balance large stretchability and high piezoelectric output. The mechanical properties of PDMS can be readily tuned by changing the base-to-curing-agent ratio, which alters the density of the cross-linked silicone network [32,33,34,35]. Although this method is commonly used in soft materials and stretchable electronics, its effect on the voltage response of BaTiO_3_/PDMS piezoelectric electronic skins under large deformation remains insufficiently understood. Clarifying this relationship is important for the rational design of wearable piezoelectric sensors that require both mechanical comfort and reliable signal generation.

Herein, we report a BaTiO_3_/PDMS-based piezoelectric electronic skin, denoted as BPPE-skin, fabricated through a blade-coating process. The study focuses on the coupled regulation of matrix composition and piezoelectric filler loading. The PDMS base-to-curing-agent ratio was varied from 5:1 to 30:1 to adjust the mechanical properties of the elastomeric matrix, while the BaTiO_3_ loading was changed from 10 to 90 wt% to evaluate the contribution of the active piezoelectric phase. The morphology, phase structure, and vibrational characteristics of the BaTiO_3_ fillers were characterized by scanning electron microscopy, X-ray diffraction, and Raman spectroscopy. The mechanical behavior of the composite films was examined by uniaxial tensile tests, and the electromechanical output was measured under controlled compression and bending. The results show that increasing the base-to-curing-agent ratio reduces the elastic modulus and improves stretchability, but excessive matrix softening is not favorable for voltage generation. Among the tested compositions, the film with a 10:1 PDMS ratio and 90 wt% BaTiO_3_ loading exhibits the highest voltage response while maintaining good flexibility. The optimized BPPE-skin was further attached to the knee, elbow, wrist, and fingers to record voltage signals during different bending motions. Distinguishable signal patterns were obtained from different joints and bending states, indicating the potential of the proposed BPPE-skin for wearable human motion monitoring and flexible human–machine interfaces. Therefore, the scientific novelty of this work lies not in the simple use of a BaTiO_3_/PDMS piezoelectric composite, but in establishing a matrix-compliance-regulated stress-transfer strategy for piezoelectric electronic skins. This study clarifies how the PDMS matrix compliance governs mechanical deformability, stress transfer to BaTiO_3_ particles, and the resulting piezoelectric output, providing a practical design guideline for stretchable and skin-conformal self-powered sensors.

## 2. Materials and Methods

### 2.1. Materials

Barium titanate (BaTiO_3_, BTO) particles with a purity of 99.5% and a nominal particle size of approximately 3 μm were purchased from Lianshuo Biological Technology Co., Ltd. (Shanghai, China). The silicone elastomer kit, Sylgard 184, consisting of a PDMS base and curing agent, was obtained from Dow Corning (Midland, MI, USA). Conductive silicone was used as the flexible charge-collecting layer, and conductive fabric strips were used as external electrical leads. All materials were used as received without further purification.

### 2.2. Fabrication of BTO/PDMS Piezoelectric Electronic Skin

The BaTiO_3_/PDMS-based piezoelectric electronic skin, denoted as BPPE-skin, was fabricated by a blade-coating method. First, predetermined amounts of BTO particles were added into the PDMS base and mechanically mixed for 6 h to obtain a uniform BTO/PDMS slurry. The BTO mass fractions were set as 10, 30, 50, and 90 wt%, calculated according to the total mass of BTO particles and PDMS precursor. Subsequently, the PDMS curing agent was added into the slurry according to different base-to-curing-agent mass ratios of 5:1, 10:1, 15:1, 20:1, 25:1, and 30:1. The mixture was further stirred and degassed for 30 min to remove trapped air bubbles.

The freshly prepared BTO/PDMS precursor was cast into thin films using a precision film applicator with a blade gap of 120 μm. The coated films were cured on a constant-temperature heating plate at 100 °C for 20 min and then naturally cooled to room temperature. After curing, a thin layer of conductive silicone was coated onto the surface of the BTO/PDMS composite film using a film applicator with a gap of 20 μm to form the charge-collecting electrode. Conductive fabric strips were then integrated on both sides of the device to serve as external leads. The obtained flexible device was used for subsequent poling and electromechanical measurements.

### 2.3. Electrical Poling

The fabricated BPPE-skin was polarized using an air corona poling system to activate its piezoelectric response. The poling process was carried out at 120 °C under a constant DC bias voltage of 1.78 kV for 1 h. After poling, the applied electric field was maintained during natural cooling to room temperature to stabilize the polarized state of the composite film. Before electrical measurements, the upper and lower electrodes were short-circuited briefly to release residual surface charges.

### 2.4. Material Characterization and Mechanical Testing

The morphology of the BTO particles was observed using a field-emission scanning electron microscope (FE-SEM, SU8010, Hitachi High-Technologies Corporation, Tokyo, Japan). The crystalline phase of the BTO particles was analyzed using an X-ray diffractometer (DX-2700X, Dandong Fangyuan Instrument Co., Ltd., Dandong, China). Raman spectra were recorded using a Renishaw inVia Raman spectrometer (Renishaw plc, Gloucestershire, UK) with a 532 nm laser excitation source.

The mechanical properties of pure PDMS and BTO/PDMS composite films were measured using a servo-driven tensile tester (AI-3000S, Gotech Testing Machines Inc., Taichung, Taiwan, China). The samples were punched into standard dog-bone-shaped specimens. The effective gauge thickness, length, and width of the narrow stretching region were 0.12 mm, 10 mm, and 2 mm, respectively. The tensile tests were performed at a constant crosshead speed of 100 mm/min. The elastic modulus was obtained from the initial linear region of the stress–strain curve, and the elongation at break was determined from the fracture strain. Each group was tested using three parallel specimens, and the average value was used for comparison.

### 2.5. Electromechanical Output Measurement

The dynamic electromechanical response of the BPPE-skin was evaluated using a linear-motor-based testing system. The voltage output was recorded synchronously using a Keithley 2611B SourceMeter/electrometer (Keithley Instruments, Inc., Solon, OH, USA). During the periodic compression tests, the applied force was controlled within 0–50 N by the linear motor and calibrated using a force gauge. Unless otherwise specified, the loading frequency was set to 1 Hz. For bending tests, the samples were subjected to repeated bending deformation under controlled bending angles, and the generated voltage signals were recorded in real time.

To investigate the effect of PDMS matrix composition, BTO/PDMS films with different base-to-curing-agent ratios were tested under identical mechanical loading conditions. To evaluate the influence of piezoelectric filler loading, samples with different BTO mass fractions were tested at a fixed PDMS ratio. All samples used in the comparison were prepared with the same electrode configuration and measured using the same electrical connection mode.

### 2.6. Human Motion Monitoring

For wearable motion monitoring, the optimized BPPE-skin with a 10:1 PDMS base-to-curing-agent ratio and 90 wt% BTO loading was attached to different human joints, including the knee, elbow, wrist, and fingers. The sensor was fixed onto the skin surface using medical adhesive tape to maintain conformal contact during joint movement. The output voltage signals generated during repeated bending and extension were collected using the Keithley 2611B SourceMeter/electrometer and recorded by a computer-based data acquisition system.

Finger bending tests were conducted at bending angles of 30°, 60°, and 90°. For knee, elbow, and wrist motion monitoring, repeated movements, including leg raising, squatting, elbow flexion, and wrist bending, were performed. Each motion was repeated five times to evaluate the repeatability of the output signals. The voltage–time curves obtained from different joint positions and bending states were compared to assess the ability of the BPPE-skin to distinguish different human motion patterns.

## 3. Results and Discussion

### 3.1. Fabrication and Material Characterization of the BPPE-Skin

**Figure 1:**
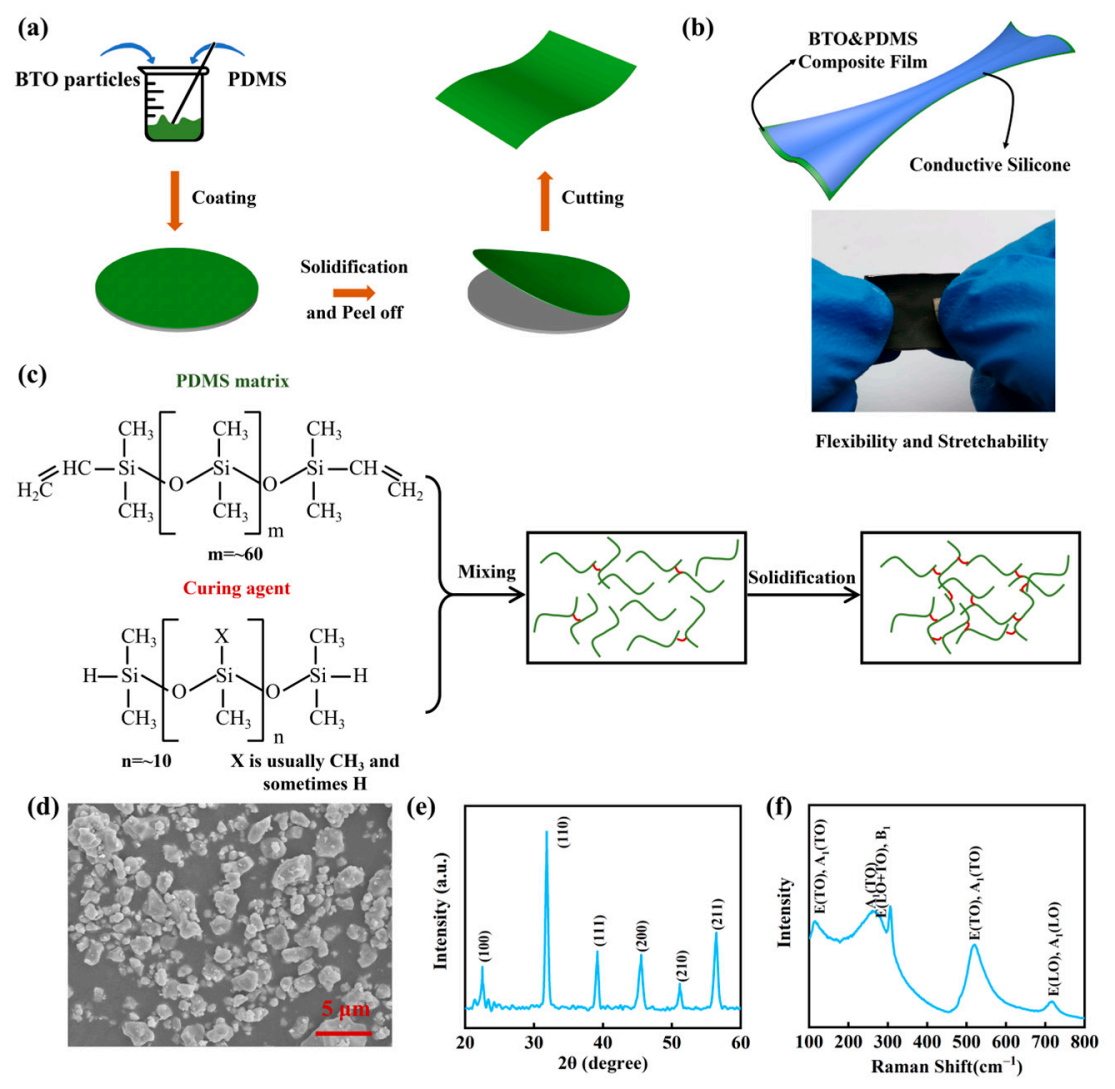
Structure, material and characterization. (**a**) Fabrication process; (**b**) schematic diagram; (**c**) the scheme of hydrosilylation reaction; (**d**–**f**) SEM image, XRD pattern and Raman spectrum of BTO powders, respectively.

Figure 1 summarizes the preparation route and basic material features of the BaTiO_3_/PDMS piezoelectric electronic skin. The device was fabricated by dispersing BTO particles into the PDMS base, followed by the addition of the curing agent and blade coating of the mixed precursor. After curing, conductive silicone was coated onto the composite film as the charge-collecting layer, and conductive fabric strips were introduced as external leads. This layered structure integrates the piezoelectric filler, stretchable polymer matrix, and flexible electrodes into one thin-film device.

In the BPPE-skin, the BTO particles act as the electromechanically active component, whereas PDMS serves as the deformable matrix. When the film is bent or compressed, the polymer network transmits part of the applied stress to the embedded ceramic particles, producing piezoelectric charges. The conductive silicone layer then collects the generated charges and transfers the signal through the fabric leads. Since the main structural components are soft, the device can maintain contact with curved surfaces during deformation, which is necessary for subsequent on-body motion tests.

The PDMS network is formed through a platinum-catalyzed hydrosilylation reaction between vinyl groups in the PDMS base and Si–H groups in the curing agent (Figure 1c). Varying the base-to-curing-agent ratio changes the relative amount of curing agent and therefore modifies the stiffness and deformability of the matrix. This point is relevant to the present study because the matrix should be soft enough for skin attachment while still allowing efficient mechanical loading of the BTO particles [36].

The BTO powder was characterized before device fabrication. The SEM image in Figure 1d shows irregular particles with micrometer-scale dimensions. The XRD pattern in Figure 1e presents the characteristic diffraction peaks of BaTiO_3_, confirming the perovskite structure of the filler [37]. Raman spectroscopy gives further information on the phase and lattice vibration of BTO. As displayed in Figure 1f, the peaks located near 261, 306, 520, and 716 cm^−1^ can be assigned to typical BaTiO_3_ vibration modes. The signal near 306 cm^−1^ is usually related to the tetragonal phase, which is beneficial for piezoelectric activity [38,39]. These results verify that the selected BTO particles are appropriate active fillers for constructing the BPPE-skin.

### 3.2. Mechanical Properties of the BTO/PDMS Composite Films

**Figure 2:**
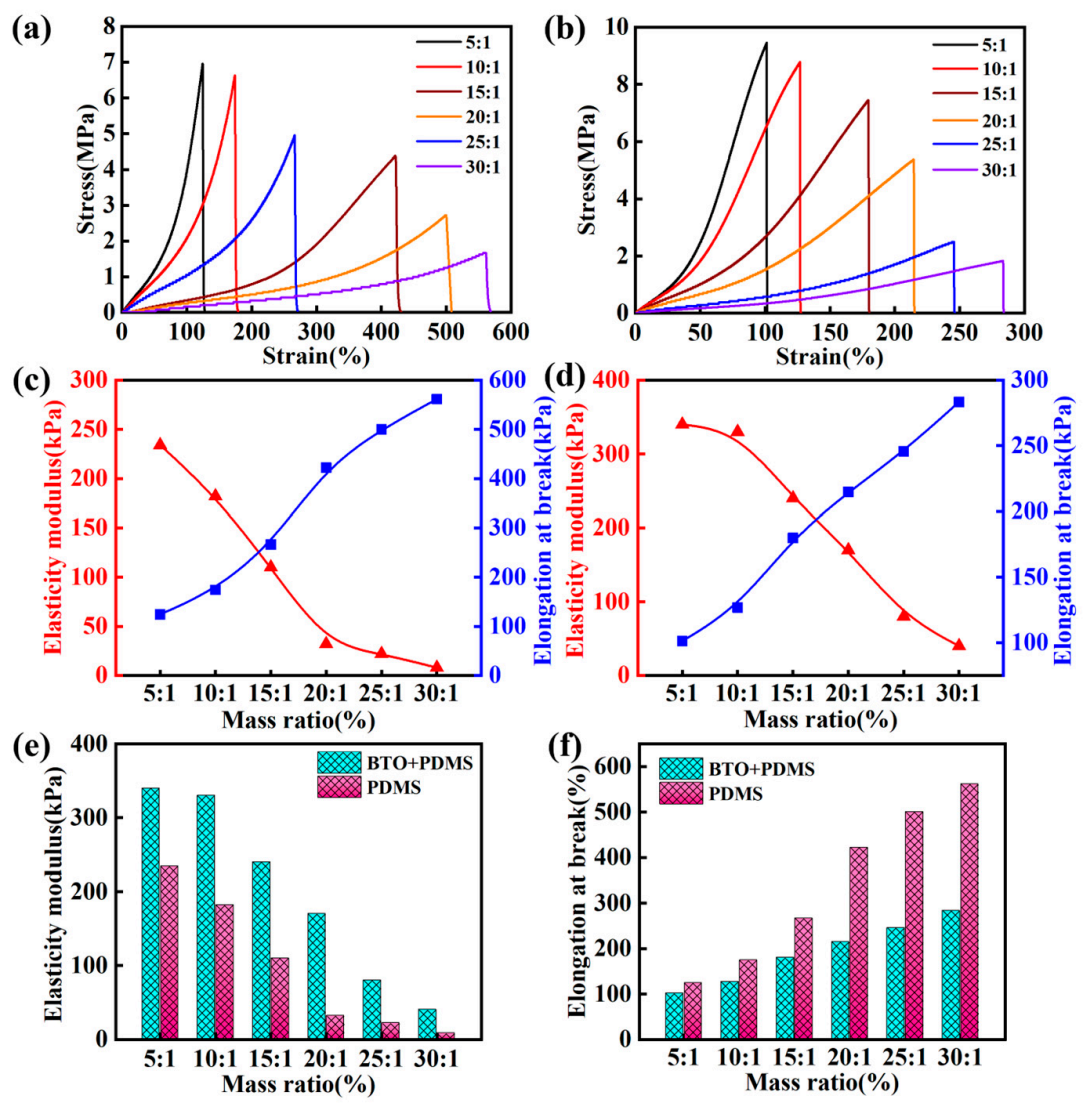
Mechanical characterization. (**a**,**b**) Uniaxial tensile stress–strain curves of the pure PDMS and BTO/PDMS composite films, respectively; (**c**,**d**) evolution of elastic modulus and elongation at break for the pure PDMS and BTO/PDMS composite films under various cross-linking degrees; (**e**,**f**) comparative analysis of the elastic modulus and elongation at break between pure PDMS and BTO/PDMS composite films at equivalent base-to-cross-linker ratios.

The mechanical behavior of the matrix and composite films was examined to evaluate whether the BPPE-skin could tolerate large deformation during skin-mounted motion sensing. Figure 2 presents the tensile properties of pure PDMS and BTO/PDMS composite films prepared with different base-to-curing-agent ratios. For both material systems, the stress–strain curves vary markedly as the ratio changes from 5:1 to 30:1. This result indicates that the mechanical properties of the films can be effectively adjusted by controlling the relative amount of curing agent.

As the base-to-curing-agent ratio increases, the curing-agent content decreases, leading to a softer PDMS network. Consequently, the elastic modulus gradually decreases, while the elongation at break increases. This trend can be explained by the reduced density of cross-linked silicone chains. A lower degree of network constraint allows the polymer chains to move and extend more easily under tensile loading, giving the film higher deformability. In contrast, samples with a lower base-to-curing-agent ratio contain more curing agent and therefore show higher stiffness but limited stretchability.

The introduction of BTO particles changes the mechanical response of the PDMS matrix. Compared with pure PDMS prepared at the same ratio, the BTO/PDMS composite films exhibit a higher elastic modulus, showing the reinforcing effect of the rigid ceramic fillers. At the same time, the elongation at break decreases after BTO incorporation. This reduction is mainly associated with the restricted movement of polymer chains and local stress concentration near the particle–matrix interface during stretching. Even so, the composite films still retain considerable deformability, especially at higher base-to-curing-agent ratios, where the elongation at break approaches 300%.

These results show that the BTO/PDMS films maintain a balance between mechanical flexibility and structural integrity. For wearable piezoelectric electronic skins, this balance is necessary because the sensing layer must deform with the skin while still transferring sufficient mechanical stress to the embedded piezoelectric particles. Therefore, the mechanical characterization provides the basis for the subsequent optimization of electromechanical output.

### 3.3. Electromechanical Output Optimization

The finite element simulation was based on the coupled mechanical and piezoelectric equations. Under quasi-static loading, the mechanical equilibrium of the composite can be expressed as

(1) ∇·σ+f=0

where σ is the stress tensor and f is the body force. The PDMS matrix was modeled as a linear elastic material, and its elastic modulus was varied to evaluate the effect of matrix compliance on deformation and stress transfer. For the BaTiO_3_ piezoelectric phase, the linear piezoelectric constitutive equations were adopted:

(2) $$\sigma =c^{E}\varepsilon -e^{T}E$$

(3) $$D=e\varepsilon +\varepsilon^{s}E$$

where $c^{E}$, ε, e, E, D, and $\varepsilon^{s}$ represent the elastic stiffness matrix, strain tensor, piezoelectric stress coefficient matrix, electric field, electric displacement, and dielectric permittivity matrix, respectively. The electric field was obtained from the electric potential by E = −∇φ, and the electric displacement satisfied ∇D = 0. These relationships describe the coupling among applied pressure, matrix compliance, stress transfer, and piezoelectric potential generation.

**Figure 3:**
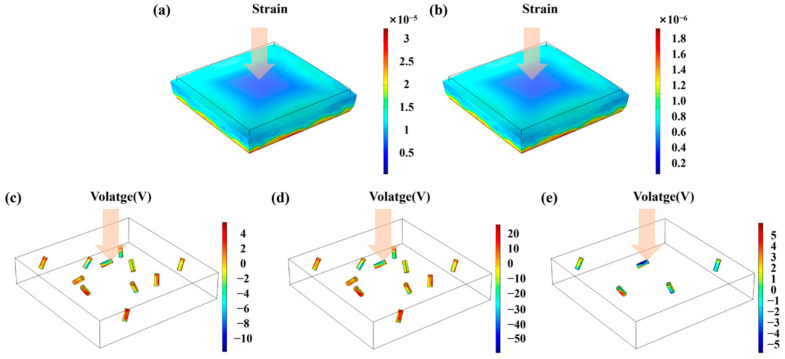
Finite element analysis (FEA) of the developed BPPE-skin. (**a**,**b**) Correlation between compressive strain and matrix elastic modulus. (**c**–**e**) Dependence of piezoelectric output performance on applied pressure and BTO filling ratio.

The electromechanical response of the BPPE-skin was first analyzed by finite element simulation to understand how the matrix and filler phase affect stress transfer and voltage generation. As presented in Figure 3a,b, the composite model was subjected to the same external pressure while the elastic modulus of the polymer matrix was changed. The strain distribution shows that a softer matrix undergoes larger deformation under identical loading, whereas a matrix with a higher modulus exhibits a smaller strain response. This result indicates that matrix stiffness plays an important role in determining how mechanical deformation is distributed inside the composite film. Although a soft matrix is beneficial for flexibility and skin conformability, excessive softness may reduce the effective transfer of stress to the piezoelectric particles because part of the mechanical energy can be accommodated by the polymer network.

The simulated piezoelectric potential distributions in Figure 3c–e further illustrate the influence of mechanical loading and BTO content on electrical output. Under larger deformation or higher applied pressure, the stress acting on the BTO domains increases, leading to a stronger piezoelectric potential. In addition, when the filler content is increased, more piezoelectric particles participate in electromechanical conversion, and the simulated output is enhanced accordingly. These simulation results provide a qualitative basis for the experimental optimization of the PDMS base-to-curing-agent ratio and BTO loading.

**Figure 4:**
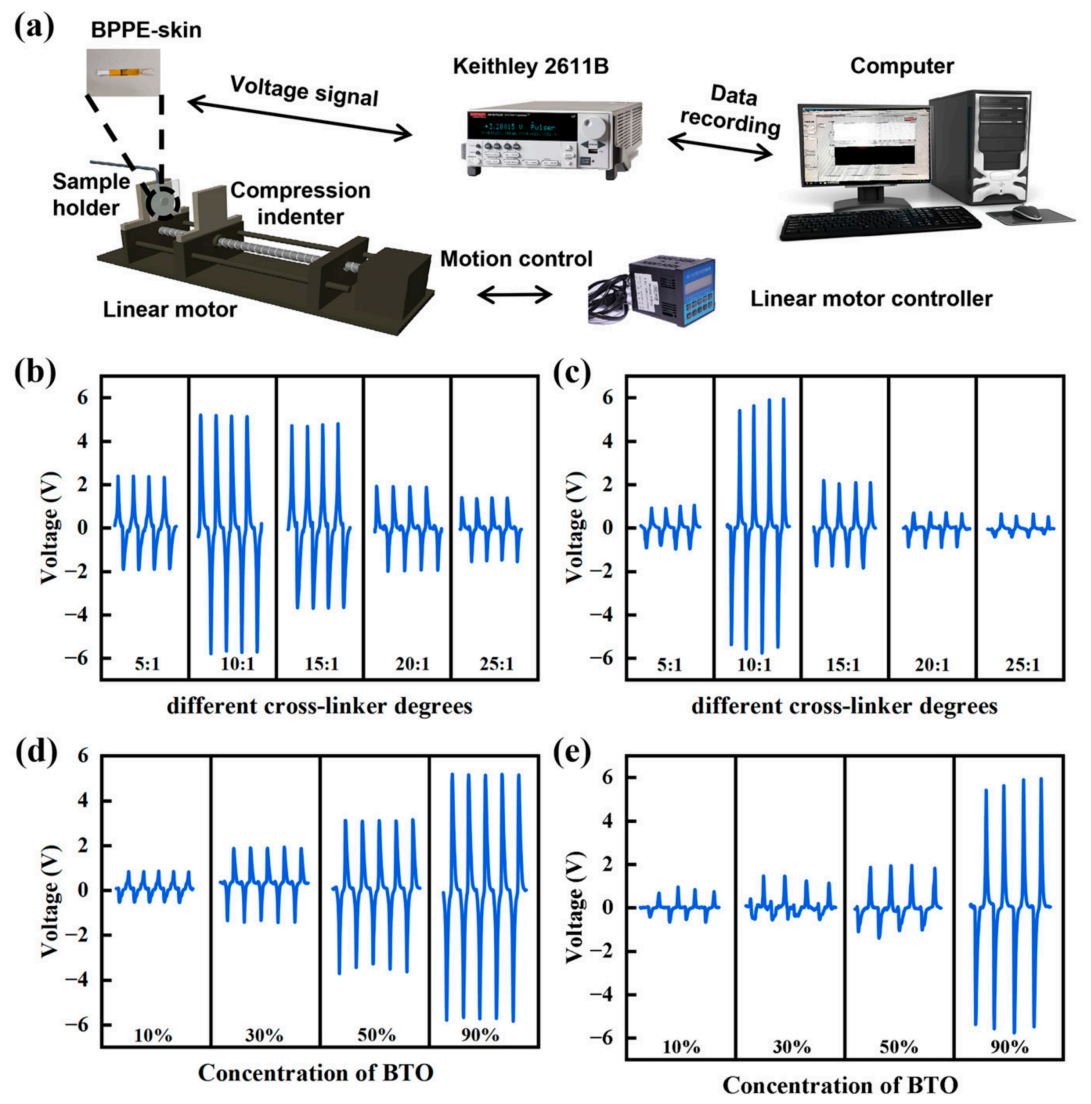
(**a**) Linear motor test system; (**b**) V-t curves under different cross-linker ratios during pressing; (**c**) V-t curves under different cross-linker ratios during bending; (**d**) V-t curves under different BTO concentrations during pressing; (**e**) V-t curves under different BTO concentrations during bending.

The electrical output of the fabricated BPPE-skin was measured using a linear-motor-based test system, as shown in Figure 4. Figure 4a schematically illustrates the electromechanical testing configuration, where the BPPE-skin was fixed on a flat rigid substrate and cyclic normal loading was applied to the active sensing area by a compression indenter. The generated voltage signal was collected from the two electrode terminals using a Keithley 2611B. Under external compression, the PDMS matrix deformed and transferred stress to the embedded BaTiO_3_ particles, thereby inducing piezoelectric charges and generating voltage output. The voltage signals obtained from samples with different PDMS base-to-curing-agent ratios are compared in Figure 4b,c. The output does not increase monotonically with the ratio. Instead, the sample with a 10:1 ratio shows the strongest voltage response among the tested groups. For the 5:1 sample, the higher stiffness of the matrix may limit deformation and reduce the effective mechanical loading transferred to the BTO particles. When the ratio is further increased beyond 10:1, the matrix becomes softer and more stretchable, but the reduced stiffness may weaken stress transfer during dynamic deformation. Therefore, the 10:1 ratio provides a more suitable balance between matrix deformability and stress transfer efficiency, resulting in a higher electromechanical output.

The effect of BTO loading was also investigated, as shown in Figure 4d,e. With the increase in BTO mass fraction from 10 to 90 wt%, the voltage amplitude increases gradually. This trend can be attributed mainly to the increased amount of active piezoelectric phase in the composite film. A higher BTO content introduces more electromechanically active particles, allowing more piezoelectric charges to be generated under the same mechanical stimulus. Meanwhile, interfacial polarization between the ceramic particles and the PDMS matrix may also contribute to the enhanced electrical response. However, the improvement should still be considered together with the mechanical properties of the composite, because excessive filler loading can affect film flexibility and particle dispersion.

Overall, the simulation and experimental results consistently show that both the PDMS matrix composition and BTO loading strongly influence the voltage output of the BPPE-skin. Among the tested samples, the composite film with a 10:1 PDMS base-to-curing-agent ratio and 90 wt% BTO loading exhibits the most favorable voltage response. This optimized composition was therefore selected for the following wearable human motion monitoring tests.

In addition, the hysteresis behavior, frequency-dependent response, long-term durability, and device-to-device reproducibility of the optimized BPPE-skin were further evaluated, as shown in Figures S1–S4. The device exhibited low hysteresis coefficients of 3.78% and 4.04% in the first and third loading-unloading tests, maintained stable periodic voltage signals at 1–3 Hz, retained over 95% of its initial output voltage after 10,000 loading cycles, and showed a device-to-device relative standard deviation of 4.56% under the same bending condition. These results further support its repeatability, dynamic response capability, long-term durability, and reliability for wearable motion monitoring.

### 3.4. Wearable Human Motion Monitoring

The optimized BPPE-skin was further evaluated for wearable human motion monitoring. Since human joints involve repeated bending, stretching, and local compression during movement, a wearable piezoelectric sensor should maintain stable contact with the skin while producing distinguishable electrical signals. In this section, the BTO loading was fixed at 90 wt%, and sensors with different PDMS base-to-curing-agent ratios were attached to several body joints to compare their response characteristics under practical motion conditions.

**Figure 5:**
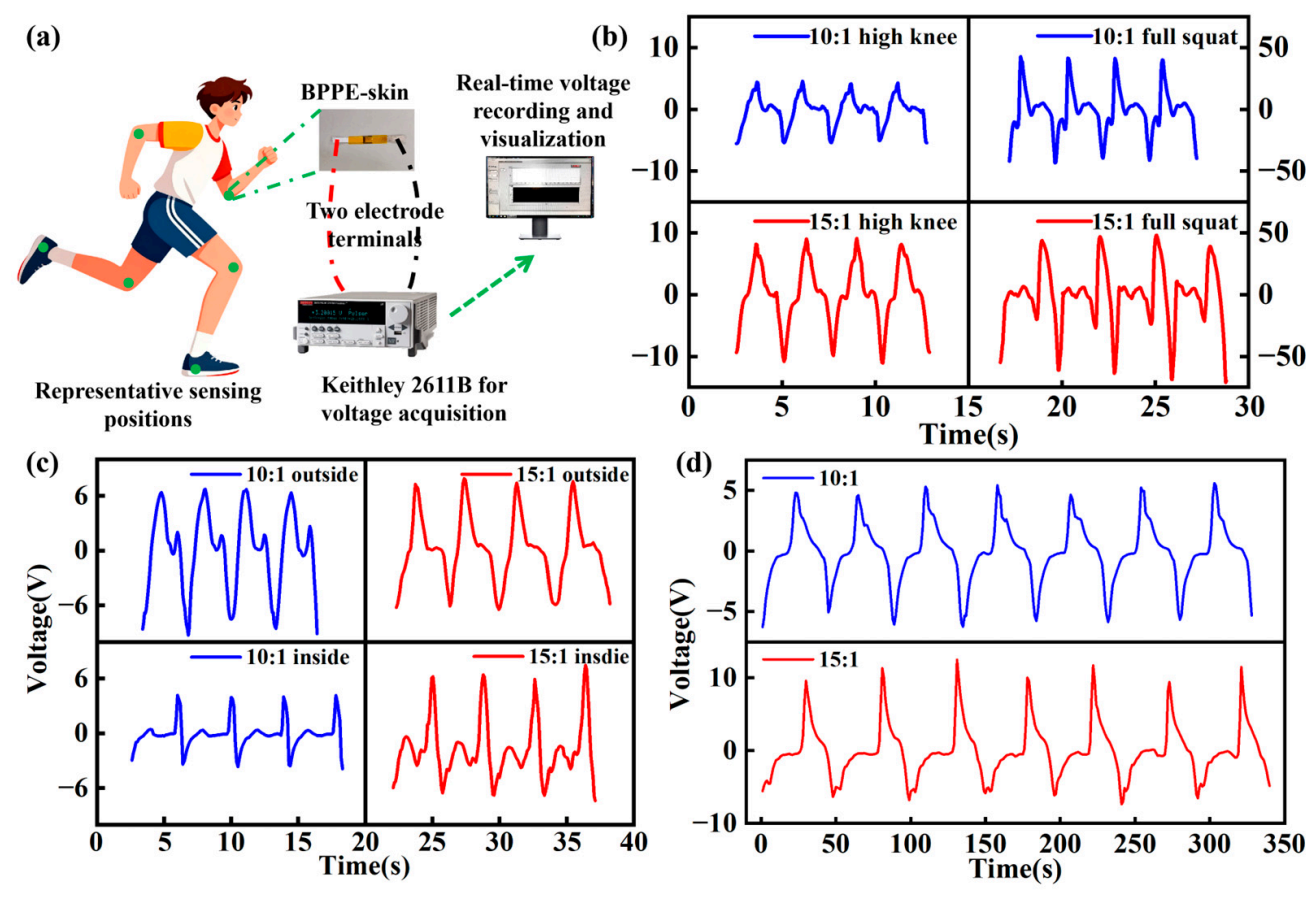
Human motion monitoring of the BPPE-skin sensor. (**a**) Schematic of the motion test system deployed to collect pressure-induced voltage signals during human movements. (**b**) V-t curves of signals collected at the knee joint during different movements. (**c**) V-t curves of signals picked up inside and outside the elbow joint. (**d**) V-t curves of signal picked up at the wrist.

Figure 5a shows the voltage acquisition schematic for human motion monitoring, in which the BPPE-skin was attached to representative sensing positions and connected to the Keithley 2611B through two electrode terminals, with the generated voltage signals recorded on a computer in real time. As shown in Figure 5b, when the 10:1 sensor was attached to the knee joint, repeated high leg raising and squatting produced periodic voltage signals with clear waveform features. The two motion modes led to different signal shapes and amplitudes, indicating that the sensor could respond to changes in joint deformation during different lower-limb movements. In comparison, the 15:1 sensor generated less distinguishable waveforms under similar motions, and the output traces contained more unstable fluctuations. This difference suggests that the matrix composition affects not only the output level under controlled loading but also the signal quality during on-body deformation.

The elbow joint was also used to examine the influence of attachment position. As shown in Figure 5c, sensors mounted on the inner and outer sides of the elbow produced different voltage responses during flexion. For the 10:1 sample, the outer elbow position generated larger and more regular voltage peaks than the inner side, which can be attributed to the greater tensile and compressive deformation experienced by the outer region during bending. The inner side showed a weaker response because the local deformation mode and strain amplitude were different. These results indicate that the BPPE-skin can capture position-dependent mechanical changes around the joint. Figure 5d further presents the wrist-bending response. The repeated voltage peaks show that the sensor can follow cyclic wrist movement and generate time-resolved electrical signals during continuous bending and extension.

**Figure 6:**
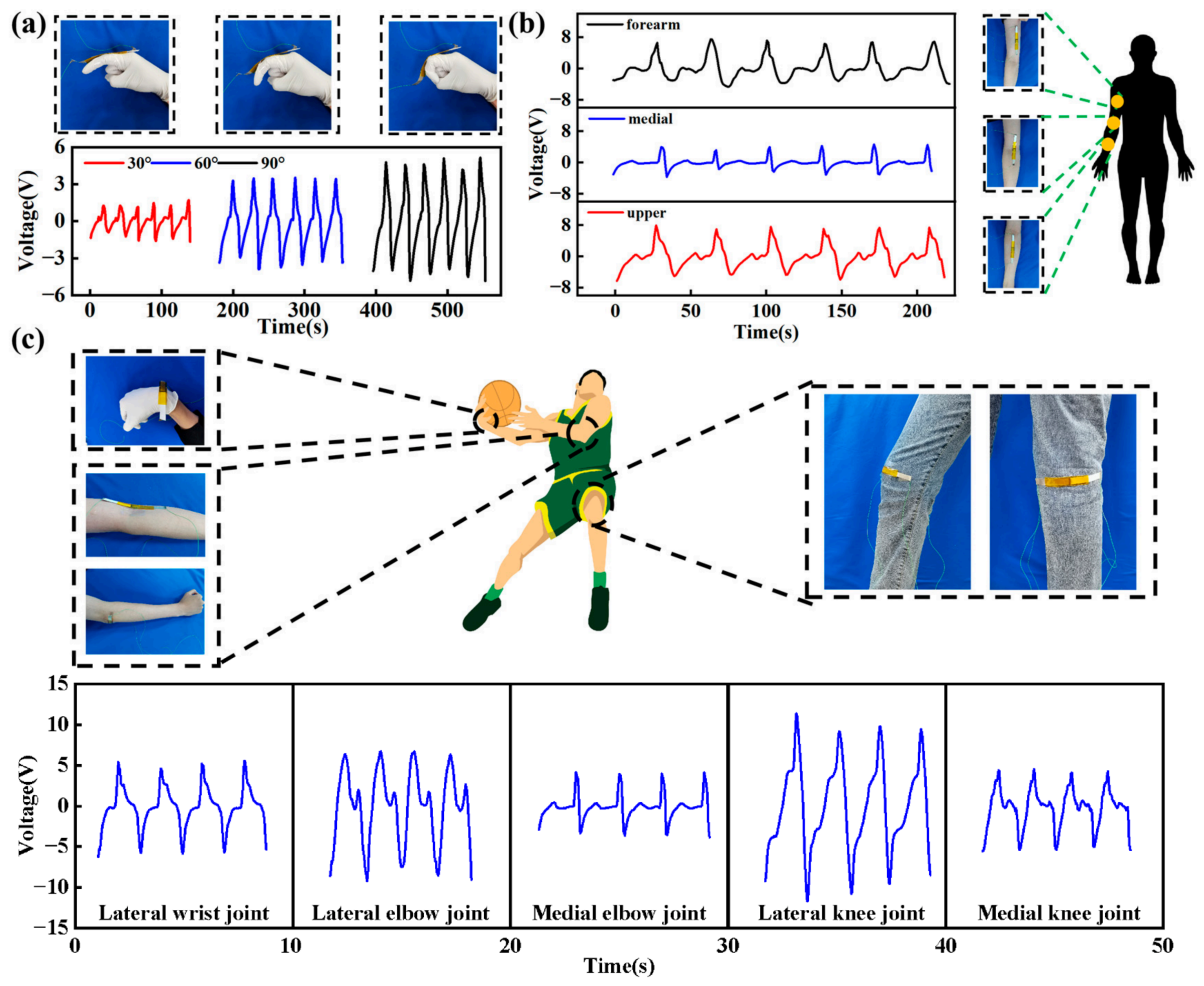
Biomechanical sensing and motion discrimination of the optimized BPPE-skin configuration. (**a**) Time-resolved voltage signatures of the knuckle-mounted sensor under incremental bending degrees. (**b**) Site-specific electrical responses yielded by fixing the device onto the upper arm, elbow joint, and forearm regions. (**c**) High-fidelity voltage-time profiles reflecting the sensor’s adaptability across multiple human articulatory pathways.

To further verify the applicability of the optimized device, the BPPE-skin with a 10:1 PDMS ratio and 90 wt% BTO loading was used in more detailed motion tests, as shown in Figure 6. Finger bending at 30°, 60°, and 90° produced gradually changing voltage responses, demonstrating that the sensor can reflect different bending degrees. A larger bending angle generally results in stronger local deformation of the attached film, leading to a higher voltage output. The periodic waveforms obtained at each angle also indicate that the sensor can respond repeatedly to small joint motions.

The sensor was then fixed at different positions of the arm, including the upper arm, elbow joint, and forearm. As shown in Figure 6b, these locations produced distinct voltage profiles during movement. This result is reasonable because different anatomical positions experience different deformation amplitudes and strain directions during joint motion. The output signal is therefore related not only to the movement type but also to the local mechanical environment of the attachment site.

Figure 6c compares the voltage signals collected from several joint regions, including the lateral wrist, lateral elbow, medial elbow, lateral knee, and medial knee. The waveforms differ in peak amplitude, waveform shape, and periodic characteristics. These differences arise from variations in joint geometry, bending range, skin deformation, and the relative position of the sensor with respect to the motion axis. Such distinguishable voltage patterns suggest that the BPPE-skin can be used to monitor different joint motions without requiring an external power supply for signal generation.

Overall, the on-body tests demonstrate that the optimized BPPE-skin can convert joint deformation into measurable voltage signals. The results also show that the 10:1 PDMS matrix provides more stable and distinguishable responses than the 15:1 sample under the tested motion conditions. Although further quantitative analysis would be needed for automatic motion classification, the present results confirm the feasibility of using the BTO/PDMS piezoelectric electronic skin for wearable human motion monitoring.

## 4. Conclusions

In this work, a stretchable and skin-conformal BaTiO_3_/PDMS-based piezoelectric electronic skin, denoted as BPPE-skin, was fabricated by a blade-coating method. The effects of the PDMS base-to-curing-agent ratio and BTO loading on the mechanical and electromechanical properties of the composite films were systematically investigated. By adjusting the PDMS ratio from 5:1 to 30:1, the elastic modulus and elongation at break of the films could be effectively tuned. Among the tested compositions, the 10:1 PDMS ratio provided the highest voltage output, indicating that appropriate matrix compliance is important for efficient stress transfer to the piezoelectric fillers. In addition, increasing the BTO mass fraction from 10 to 90 wt% enhanced the electrical output, which may be associated with the increased amount of active piezoelectric phase and interfacial polarization within the composite. The optimized BPPE-skin with a 10:1 PDMS ratio and 90 wt% BTO loading maintained good flexibility and could be conformally attached to different human joints. On-body tests on the knee, elbow, wrist, and fingers produced distinguishable voltage patterns under different bending motions, demonstrating its potential for wearable human motion monitoring and flexible human–machine interfaces. Inspired by recent advances in self-healing nanogenerators [40,41], future studies may further explore the integration of self-healing polymer networks or recoverable electrode interfaces into BPPE-skin, aiming to enhance its mechanical damage tolerance and long-term service reliability in wearable applications.

## Data Availability

Data is contained within the article and the Supplementary Materials.

## Supplementary Materials

The following supporting information can be downloaded at: https://www.mdpi.com/article/10.3390/s26154781/s1, Figure S1. Hysteresis of optimized BPPE-skin during the first and third loading-unloading tests. Figure S2. Frequency-dependent voltage response of the opti-mized BPPE-skin under cyclic loading at 1, 2, and 3 Hz. Figure S3. Long-term cyclic durability of the optimized BPPE-skin under 10,000 repeated loading cycles. Figure S4. Device-to-device reproduc-ibility of three independently fabricated optimized BPPE-skin devices under the same bending condition.
