# Supplementary material for: Ultra-Stretchable and Skin-Conformal Piezoelectric Electronic Skin for Self-Powered Human Motion Monitoring
Source: Sensors (Basel). 2026 Jul 28;26(15):4781. doi: 10.3390/s26154781 (PMC13469370; doi:10.3390/s26154781)
Supplement: Supplementary file 1 [file sensors-26-04781-s001.zip › sensors-4392331-supplementary.pdf]

Supporting Information

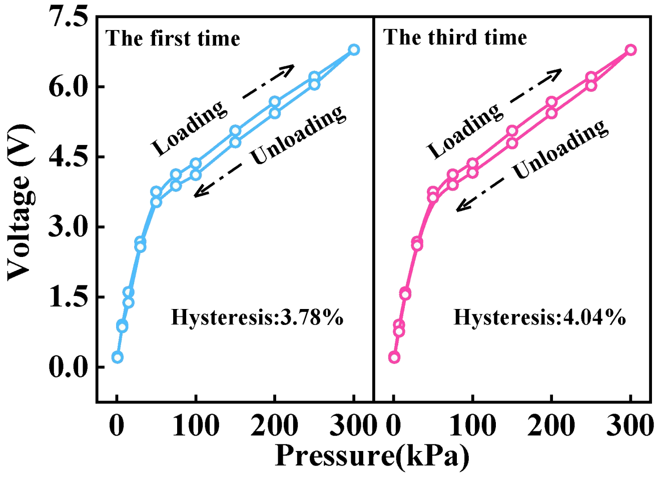

Figure S1. Hysteresis of optimized BPPE-skin during the first and third loading-unloading tests.

Figure S1 shows the hysteresis behavior of the optimized BPPE-skin under repeated loading-unloading tests. The hysteresis coefficients of the first and third tests were 3.78% and 4.04%, respectively. The slight increase may be related to the viscoelastic relaxation of the PDMS matrix, while the overall hysteresis remained below 5%, indicating relatively low hysteresis and good repeatability.

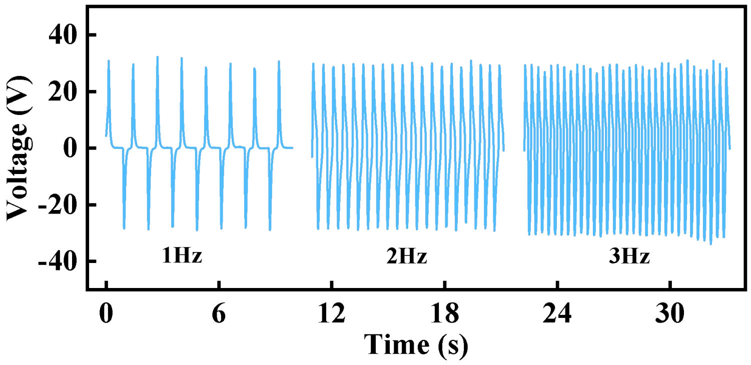

Figure S2. Frequency-dependent voltage response of the optimized BPPE-skin under cyclic loading at 1, 2, and 3 Hz.

Figure S2 shows the frequency-dependent voltage response of the optimized BPPE-skin under cyclic loading at 1, 2, and 3 Hz. The device generated stable and periodic voltage signals at all tested frequencies. As the loading frequency increased, the voltage peaks became denser due to the shortened loading period, while the output signals remained clearly distinguishable. These results indicate that the optimized BPPE-skin can reliably respond to cyclic deformation within the tested frequency range.

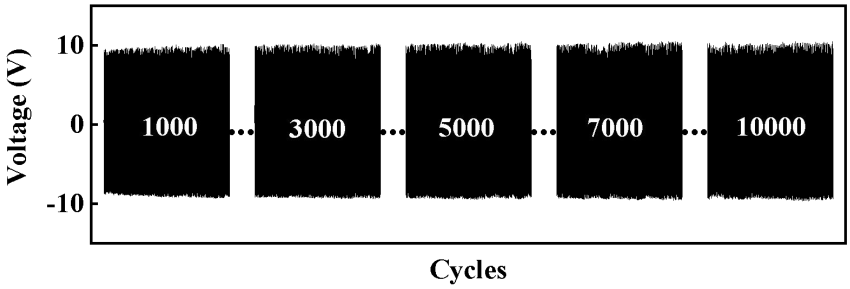

Figure S3. Long-term cyclic durability of the optimized BPPE-skin under 10,000 repeated loading cycles.

Figure S3 shows the long-term cyclic durability of the optimized BPPE-skin under repeated mechanical loading. Representative voltage signals collected after 1000, 3000, 5000, 7000, and 10,000 cycles remained stable and periodic, without obvious waveform distortion. After 10,000 cycles, the output voltage retained over 95% of its initial value, indicating good long-term repeatability and mechanical durability.

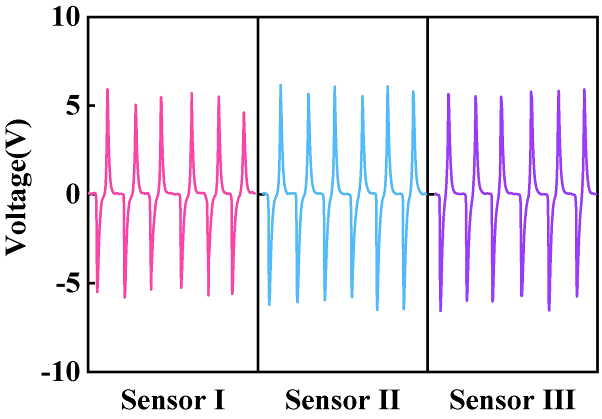

Figure S4. Device-to-device reproducibility of three independently fabricated optimized BPPE-skin devices under the same bending condition.

Figure S4 shows the device-to-device reproducibility of three independently fabricated optimized BPPE-skin devices under the same bending condition. Sensor I, Sensor II, and Sensor III all generated stable and periodic voltage signals with similar waveform features. The average positive peak voltages of Sensor I, Sensor II, and Sensor III were 5.38, 5.90, and 5.71 V, respectively, giving an overall average peak voltage of 5.66 V and a device-to-device relative standard deviation of 4.56%. These results indicate good fabrication reproducibility of the optimized BPPE-skin.
